# Supplementary material for: Long-term effects of Omicron BA.2 breakthrough infection on immunity-metabolism balance: a 6-month prospective study
Source: Nat Commun. 2024 Mar 19;15:2444. doi: 10.1038/s41467-024-46692-z (PMC10951309; doi:10.1038/s41467-024-46692-z)
Supplement: Supplementary file 1 — Supplementary Information [file 41467_2024_46692_MOESM1_ESM.pdf]

Supplemental information

Long-term effects of Omicron BA.2 breakthrough infection on immunity-metabolism balance: a 6-month prospective study

Supplementary Figures

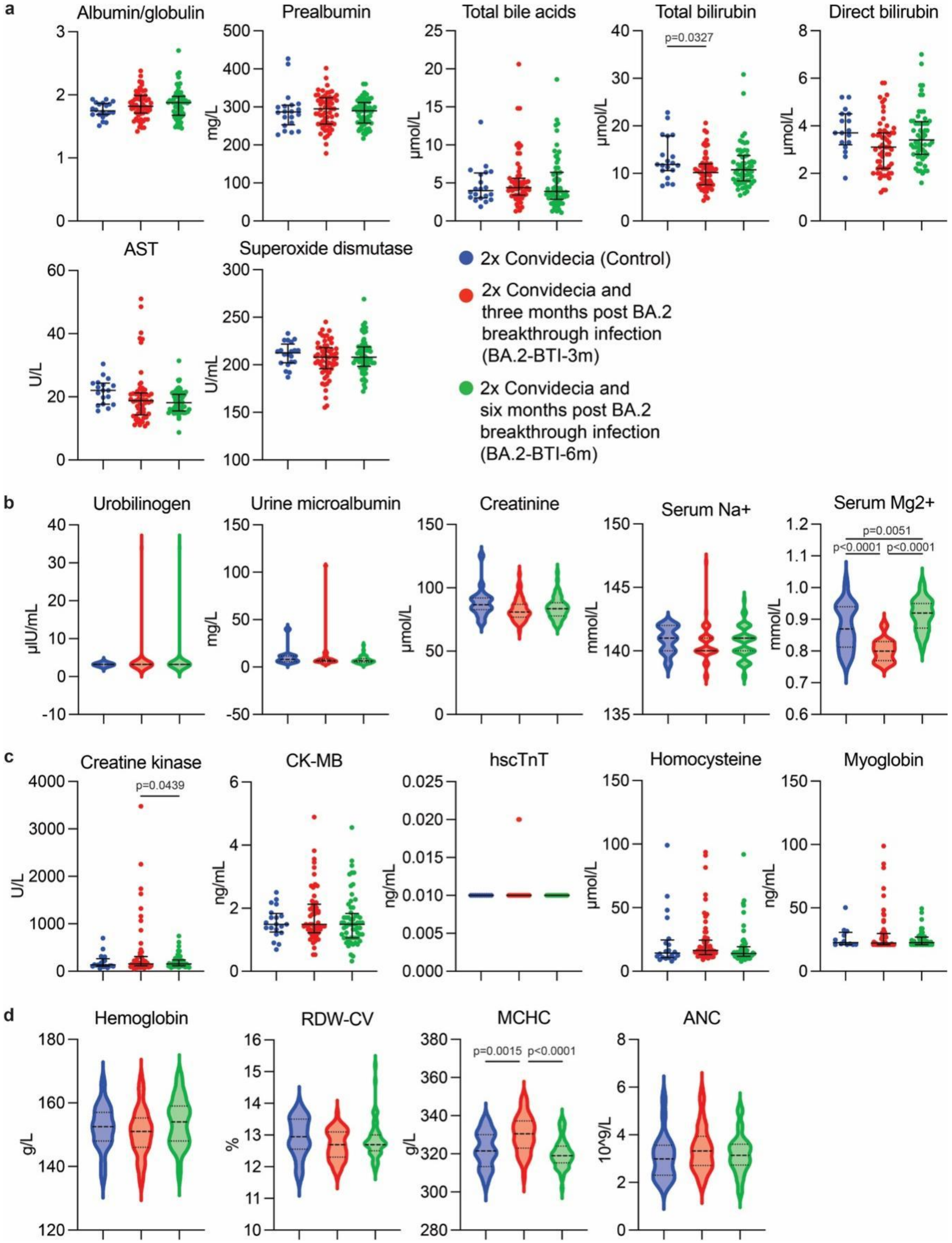

Supplementary Fig.1 Supplemented clinical parameters for assessing functions of the liver, the kidneys, the heart, and hematopoietic system after Omicron BA.2 breakthrough infection. (a) Supplementary parameters the liver function and inflammatory factors at three months post BA.2

breakthrough infection (BTI) (n=59, one participant missed this time of routine blood test, and two extra missing values in AST) and six months post BA.2 BTI (n=60), (n=20 biologically independent samples in the Control group, one missing value in Total bilirubin, and two in Direct bilirubin and AST, separately). **(b)** Parameters for the renal function (n=20 biologically independent samples in Control group, eight persons did not take the tests of Urobilinogen and Urine microalbumin; n=59 for BA.2-BTI-3m; n=60 for BA.2-BTI-6m). **(c)** Factors for myocardial function (n=20 for Control group, ten did not take the test of hscTnT, eight persons did not take the test of Myoglobin; n=59 for BA.2-BTI-3m, one missing value in Creatine kinase, three and two missing values in tests of hscTnT and Myoglobin; n=60 for BA.2-BTI-6m). **(d)** Changes in the hematopoietic parameters (n=20 for Control group; n=59 for BA.2-BTI-3m, one missing value in tests of Hemoglobin and MCHC separately; n=60 for BA.2-BTI-6m). The Control group is coloured in blue. Groups of BA.2-BTI-3m and BA.2-BTI-6m were stratified by time after BA.2 BTI on 2x Convidecia, and coloured in red and green separately. AST: aspartate aminotransferase; CK-MB: Creatine kinase MB isoenzyme; hscTnT: high-sensitivity cardiac troponin T; RDW-CV: Red blood cell distribution width - CV value; MCHC: Mean red blood cell hemoglobin concentration; ANC: Absolute neutrophil count. Convidecia: Ad5-vectored COVID-19 vaccine. Data are presented as median with interquartile range. P values reflect two-sided ordinary ANOVA tests adjusted for multiple comparisons. Source data are provided as a Source Data file.

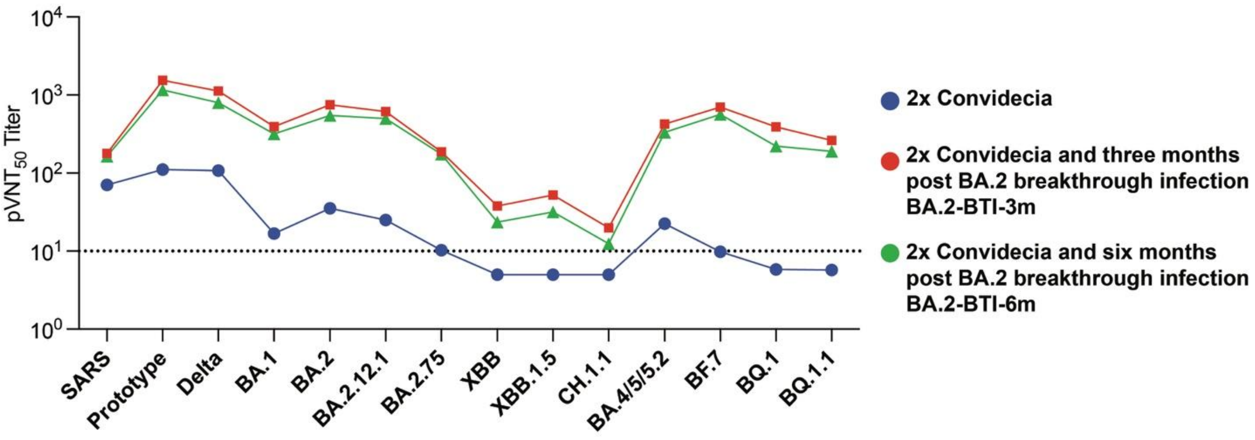

Supplementary Fig.2 BA.2 breakthrough infection elicits durable broad-neutralizing Abs against major SARS-CoV-2 variants, Omicron sub-variants and SARS-CoV.

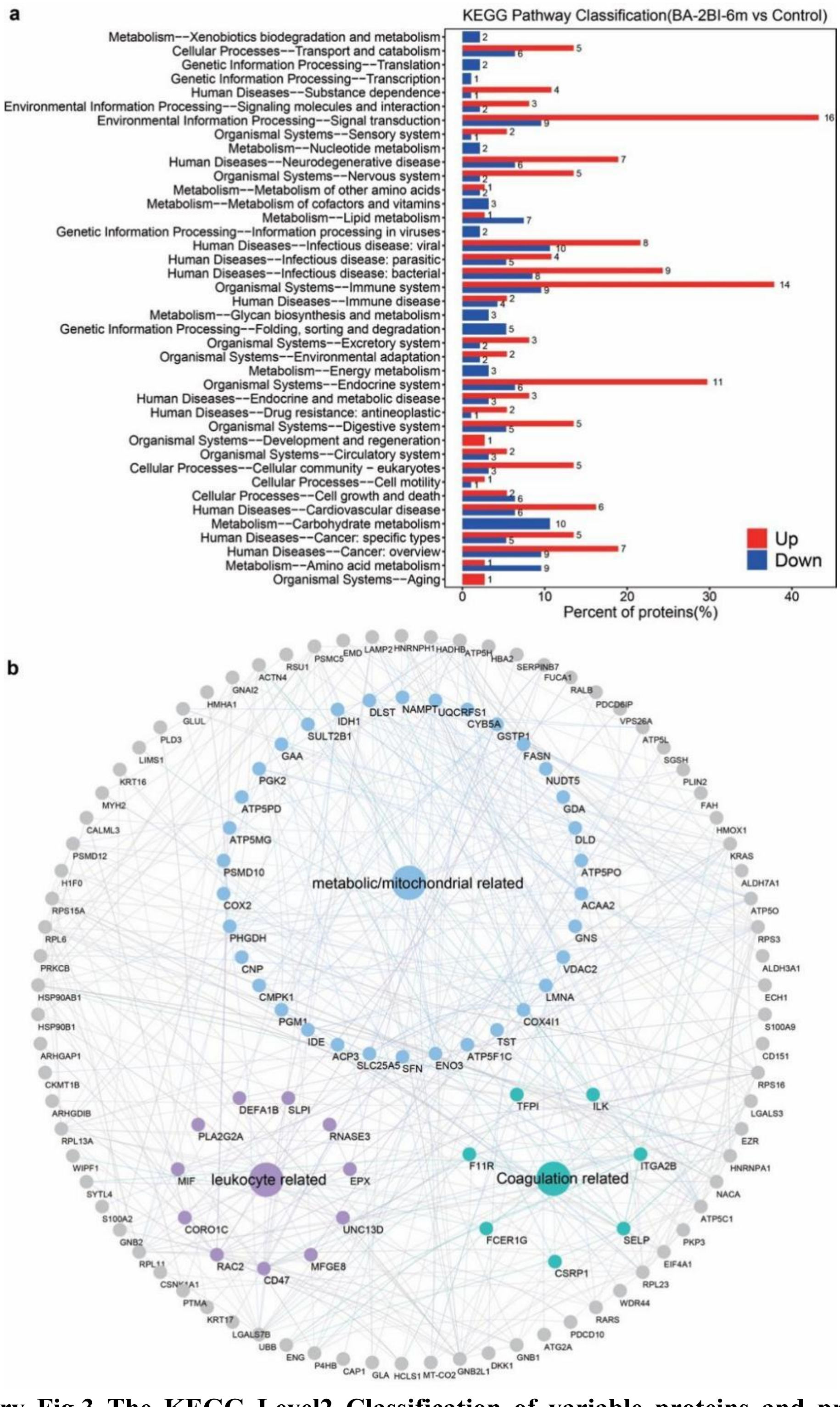

Supplementary Fig.3 The KEGG Level2 Classification of variable proteins and protein-protein interactions. (a) The distribution of variable proteins ($p<0.05$) at the KEGG Level2 of the BA.2-BTI-6m compared with the control group. The plot shows the ratio (%) of the variable proteins annotated to each Level2 KEGG pathway. **(b)** The variable protein-protein interactions. Immunity, coagulation and metabolism related proteins are labeled with different colors, other proteins that directly interact with them are labeled with gray.

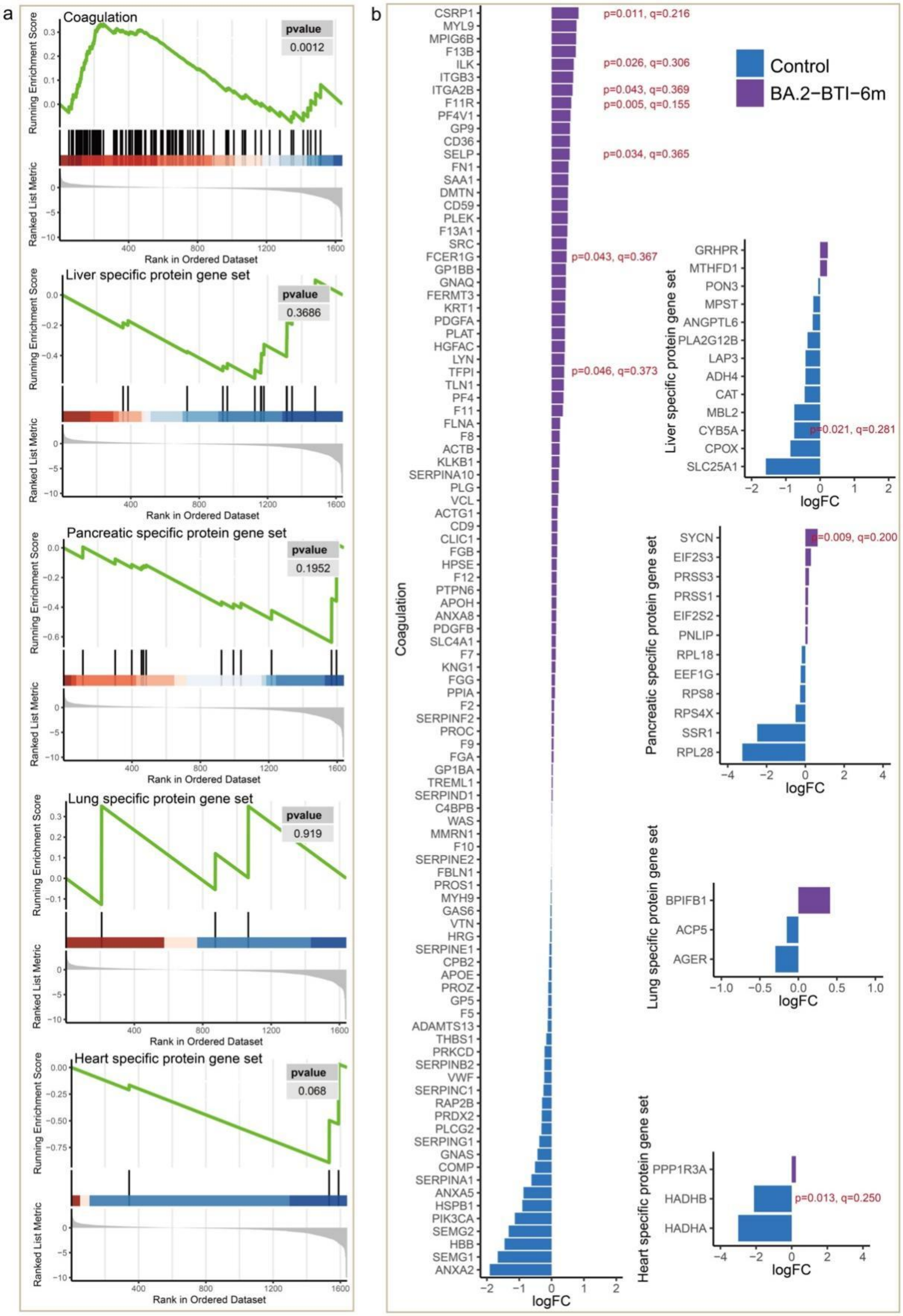

Supplementary Fig.4 GSEA analysis of tissue proteome specific genes.

(**a**) The GSEA map shows the enrichment of coagulation related genes and proteome specific genes in the liver, lung, heart, and pancreatic tissues. (**b**) The intersection and difference between coagulation related genes, liver, lung, heart, and pancreatic tissue specific genes, and measured proteins. Each column

represents the intersection of tissue specific protein gene sets and measured proteins..The list of tissue-specific proteins used for GSEA analysis in the heart, liver, lungs, and pancreas was derived from the study by Jiang et al. (Cell. 2020, DOI: 10.1016/j.cell.2020.08.036). This study constructed a quantitative map of the proteome of different tissues in the human body. P values are calculated by the two tailed Wilcoxon rank sum test. Q value adjusted by FDR.

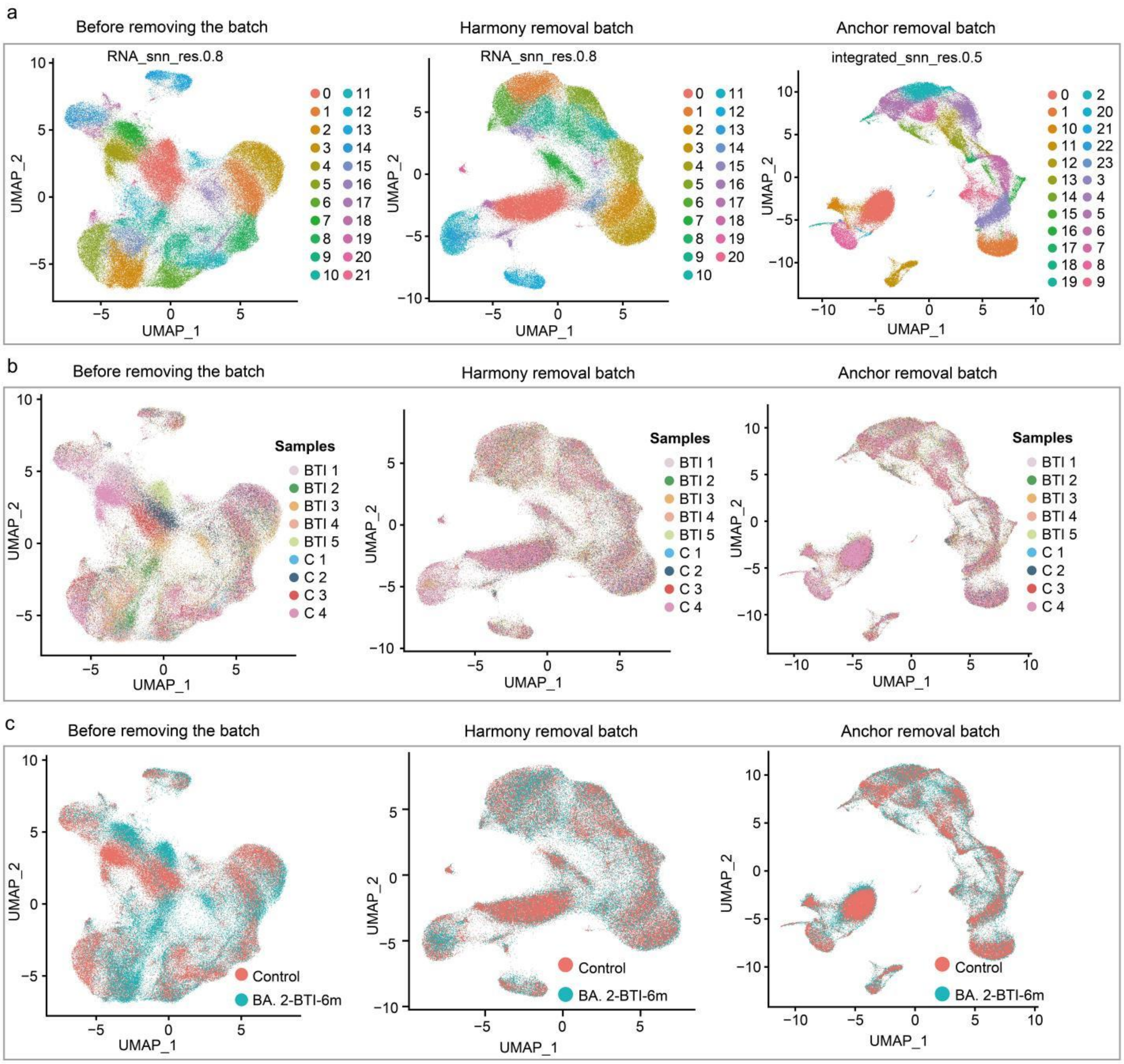

Supplementary Fig.5 Removal of individual and inter group batch effects.

(a) The UMAP graph displays cell clustering before individual and inter group batch effects removal. (**b**) The UMAP graph shows the cell clustering after removing individual and inter group batch effects using the Harmony algorithm. (**c**) The UMAP graph shows the cell clustering after removing individual and inter group batch effects using the Anchor algorithm of Seurat.

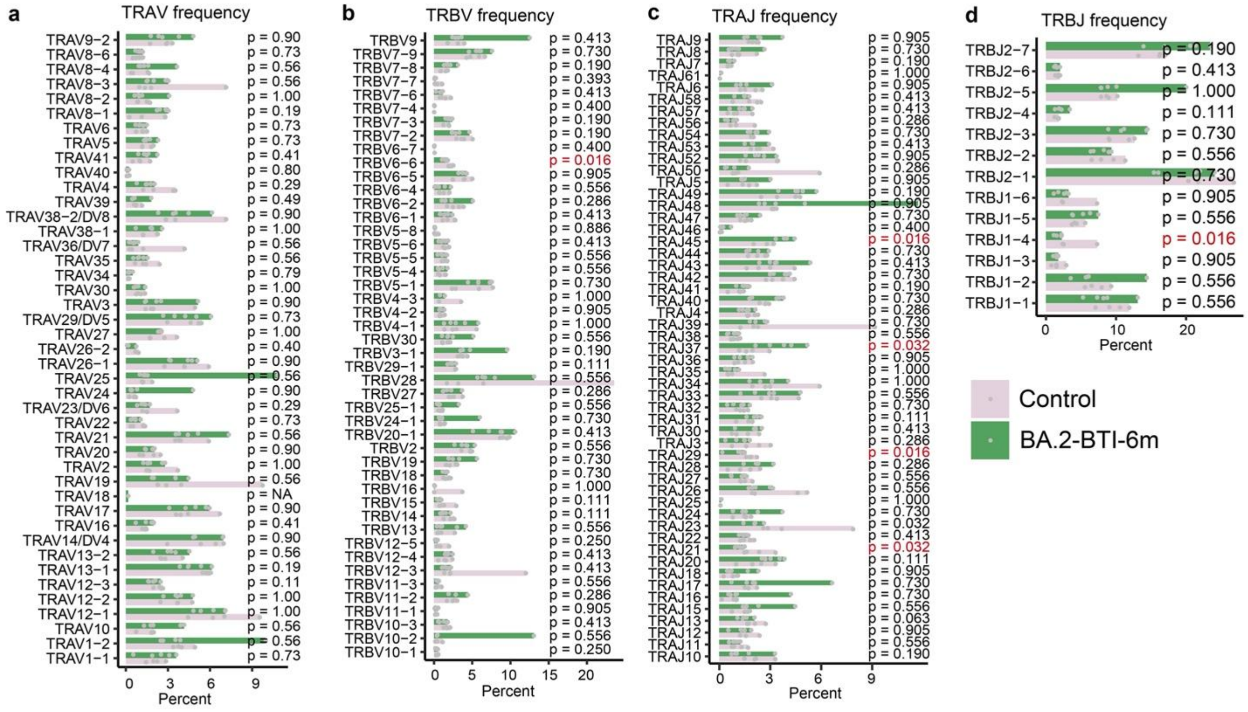

Supplementary Fig.6 Characteristics of TCR repertoire at six months post BA.2 breakthrough infection. (a) The frequency of use of the V gene in the TCRα chain. **(b)** The frequency of use of the V gene in the TCRβ chain. **(c)** The frequency of use of the J gene in the TCRα chain. **(d)** The frequency of use of the J gene in the TCRβ chain. Genes with P value less than 0.05 are marked in red. P values are calculated by the two tailed Wilcoxon rank sum test. A P-value of NA means that the gene was only detected in one of the groups.

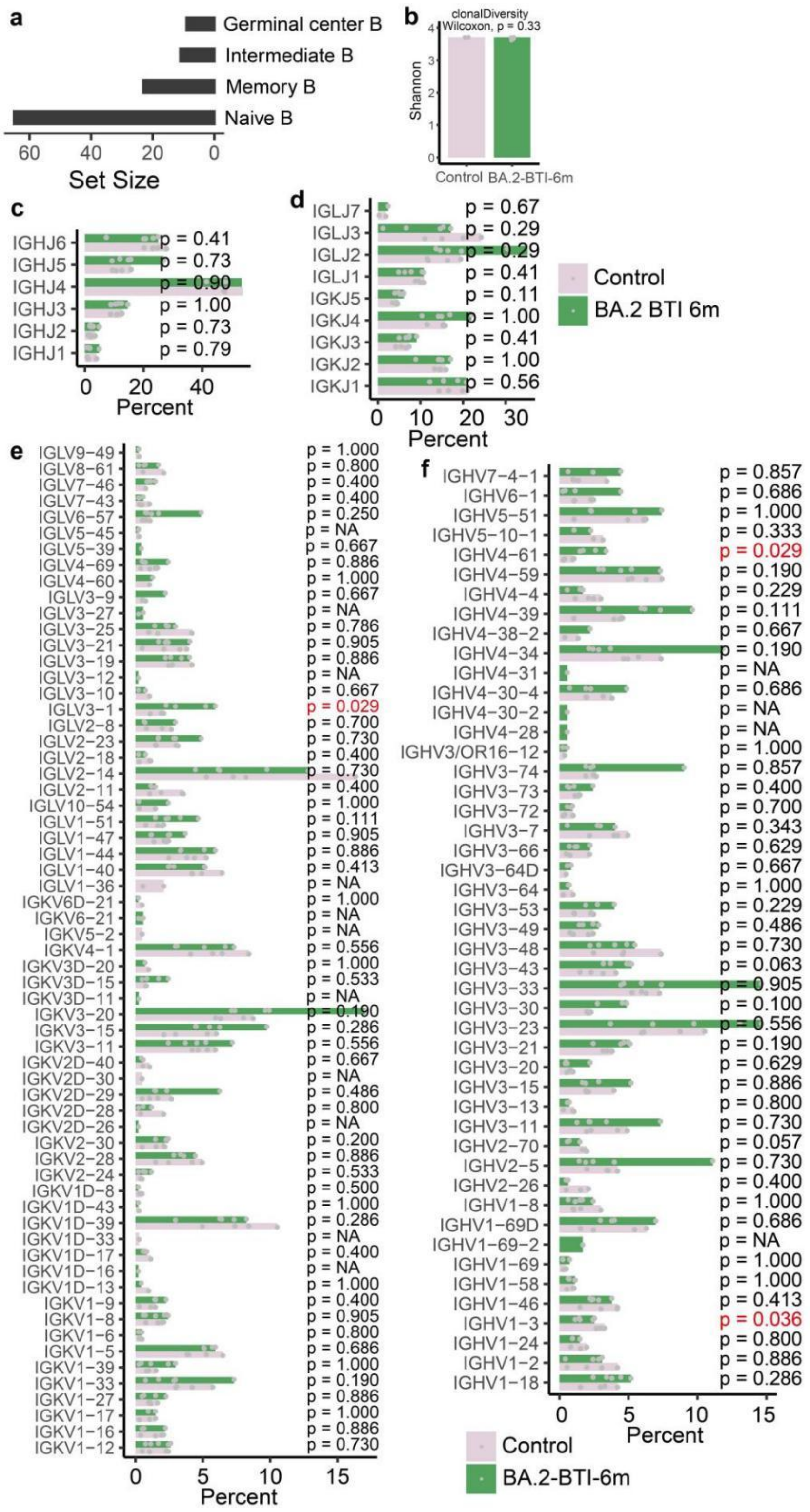

Supplementary Fig.7 Clustering of B cells and BCR repertoire at six months post BA.2 breakthrough infection.

(a) Number of differentially expressed genes in different B cell sub-clusters in the control and BA.2-BTI-6m groups. (**b**) Bar chart showing difference in BCR diversity between both groups. (**c**) The

frequency of use of the BCR heavy chain J gene. P values are calculated by the two tailed Wilcoxon rank sum test, Control (n=4), BA.2-BTI-6m (n=5). (**d**) The frequency of use of the BCR light chain J gene. (**e**) The frequency of use of the BCR light chain V gene. (**f**) The frequency of use of the BCR heavy chain V gene. Genes with P value less than 0.05 are marked in red. P values are calculated by the two tailed Wilcoxon rank sum test. A P-value of NA means that the gene was only detected in one of the groups.

Supplementary Tables.

Supplementary Table 1. General information of the participants

Groups	BA.2 breakthrough infection (BTI) groups	Control group
No. of participants	60	20
Age (median, range)	20 (18-29)	25 (20-41)
Sex (Male %)	80%	75%
Race (%)	Mongoloid (100%)	Mongoloid (100%)
(Chronic) Medical conditions	None	None
COVID-19 vaccination history	Two doses of Convidecia	Two doses of Convidecia
Interval between the 1st and the 2nd doses	12 months	12 months
Recent infection history	BA.2 BTI	··
No. of mild infection (%)	56 (93.3%)	··
Diagnosed method*	Sequencing	Qrt-PCR
Interval between the 2nd dose and BA.2 BTI	~10 months	··
Sampling time points	Three and six months post BA.2 BTI	~12 months after the 2nd shot of Convidecia
Time of day for sampling	7-9 a.m.	7-9 a.m.

*Diagnosed according to the Chinese Center for Disease Control and Prevention (CCDC) diagnosis guideline

Supplementary Table 2. Numbers of participants in each data type in different groups.

Data type	BA.2 breakthrough infection 3-mo (male)	BA.2 breakthrough infection 6-mo (male)	2x Convidecia (male)
Clinical blood parameters	57-60 (45-48)	58-60 (46-48)	10-20 (7-15)
Pseudotyped virus neutralization assays	20-60 (16-48)	20-60 (16-48)	8-20
Serum proteomics	..	10 (10)	8 (8)
Single cell transcriptomics /TCR/BCR sequencing	..	5 (5)	4 (4)
