# Supplementary material for: Long-term effects of Omicron BA.2 breakthrough infection on immunity-metabolism balance: a 6-month prospective study
Source: Nat Commun. 2024 Mar 19;15:2444. doi: 10.1038/s41467-024-46692-z (PMC10951309; doi:10.1038/s41467-024-46692-z)
Supplement: Supplementary file 3 — Description of Additional Supplementary Files [file 41467_2024_46692_MOESM3_ESM.pdf]

Description of Additional Supplementary Files

File Name: Supplementary Data 1

Description: The raw expression profile and differential analysis statistical results of all proteins. The p-value value of differential proteins is calculated using a two tailed T-test and adjusted using the fdr method.

File Name: Supplementary Data 2

Description: Differential expression genes of the main cell types between the control group and BA.2-BTI-BTI. The p-value of differentially expressed genes is calculated by the two tailed Wilcoxon rank sum test and the p-value adjustment is performed using bonferroni method based on the total number of genes in the dataset.

File Name: Supplementary Data 3

Description: Differential expression genes of four CD14 Mono clusters. The p-value of differentially expressed genes is calculated by the two tailed Wilcoxon rank sum test and the p-value adjustment is performed using bonferroni method based on the total number of genes in the dataset.

File Name: Supplementary Data 4

Description: Differential expression of genes in the main MS1 related cells of the control group and BA.2-BTI-BTI. The p-value of differentially expressed genes is calculated by the two tailed Wilcoxon rank sum test and the p-value adjustment is performed using bonferroni method based on the total number of genes in the dataset.

File Name: Supplementary Data 5

Description: Differential expression genes between amplified CD8 TEM and non amplified CD8 TEM. The p-value of differentially expressed genes is calculated by the two tailed Wilcoxon rank sum test and the p-value adjustment is performed using bonferroni method based on the total number of genes in the dataset.

File Name: Supplementary Data 6

Description: Differential expression genes of B cell subsets in the control group and BA.2-BTI-BTI. The p-value of differentially expressed genes is calculated by the two tailed Wilcoxon rank sum test and the p-value adjustment is performed using bonferroni method based on the total number of genes in the dataset.

File Name: Supplementary Data 7

Description: Abbreviations of the differentially expressed proteins in the text.
