# Supplementary material for: Long-term effects of Omicron BA.2 breakthrough infection on immunity-metabolism balance: a 6-month prospective study
Source: Nat Commun. 2024 Mar 19;15:2444. doi: 10.1038/s41467-024-46692-z (PMC10951309; doi:10.1038/s41467-024-46692-z)
Supplement: Supplementary file 5 — Reporting Summary [file 41467_2024_46692_MOESM5_ESM.pdf]

nature portfolio

Corresponding author(s): Prof. George Fu Gao, Prof. Yueyun Ma and Dr. Xin Zhao

Last updated by author(s): Feb 21, 2024

Reporting Summary

Nature Portfolio wishes to improve the reproducibility of the work that we publish. This form provides structure for consistency and transparency in reporting. For further information on Nature Portfolio policies, see our Editorial Policies and the Editorial Policy Checklist.

Statistics

For all statistical analyses, confirm that the following items are present in the figure legend, table legend, main text, or Methods section.

n/a	Confirmed	
☐	☒	The exact sample size (*n*) for each experimental group/condition, given as a discrete number and unit of measurement
☐	☒	A statement on whether measurements were taken from distinct samples or whether the same sample was measured repeatedly
☐	☒	The statistical test(s) used AND whether they are one- or two-sided<br>*Only common tests should be described solely by name; describe more complex techniques in the Methods section.*
☒	☐	A description of all covariates tested
☐	☒	A description of any assumptions or corrections, such as tests of normality and adjustment for multiple comparisons
☐	☒	A full description of the statistical parameters including central tendency (e.g. means) or other basic estimates (e.g. regression coefficient) AND variation (e.g. standard deviation) or associated estimates of uncertainty (e.g. confidence intervals)
☐	☒	For null hypothesis testing, the test statistic (e.g. *F*, *t*, *r*) with confidence intervals, effect sizes, degrees of freedom and *P* value noted<br>*Give P values as exact values whenever suitable.*
☒	☐	For Bayesian analysis, information on the choice of priors and Markov chain Monte Carlo settings
☐	☒	For hierarchical and complex designs, identification of the appropriate level for tests and full reporting of outcomes
☒	☐	Estimates of effect sizes (e.g. Cohen's *d*, Pearson's *r*), indicating how they were calculated

Our web collection on statistics for biologists contains articles on many of the points above.

Software and code

Policy information about availability of computer code

Data collection	Proteomics data: After fractionation, the peptides were lyophilized and separated using a C18 column (25 cm × 75 μm) on an EASY-nLCTM 1200 system (Thermo Fisher, Waltham, MA, USA). The flow rate was 300 nL/min and the linear gradient was set accordingly. The 4D-DIA mass spectrometry (MS) data for the library was acquired via the PASEF method as follows: MS data were collected over an m/z range of 100 to 1700, and during each MS/MS data collection, each TIMS cycle time was 1.1 s; each cycle included 1 MS and 10 MS/MS 100 msec TIMS scans; in each of the 10 PASEF MS/MS scans an average of 12 precursors were selected, resulting in an MS/MS data acquisition rate of 109 Hz. For the DIA, 56 DIA windows were acquired (automatic gain control target 3e6 and auto for injection time), and the collision energy was ramped linearly as a mobility function from 59 eV at 1/K0 = 1.6 Vs cm−2 to 20 eV at 1/K0 = 0.6 Vs cm−2. The MS/MS spectra were recorded from 100 to 1700 m/z.<br>Single-cell sequencing: the single-cell suspension was prepared in water for cDNA library amplification using the 10× Genomics Chromium Next GEM Single Cell 5′ Reagent Kits (version 2.0; Cat. No. 1000165). The Chromium™ Single Cell 5′ Library Construction Kit (Cat. No. 1000020) was used to construct the DNA library. The constructed library was then sequenced using PE150 sequencing on an Illumina Nova 6000 platform. T cell V(D)J and B cell V(D)J enrichment analyses were performed using the 10× Genomics Chromium™ Single Cell V(D)J Enrichment Kit, Human T Cell (Cat. No. 1000005) and Human B Cell (Cat. No. 1000016) according to the manufacturer's instructions. The libraries were amplified using a Chromium TCR amplification kit (Cat. No. 1000252) and BCR amplification kit (Cat. No. 1000253), and the experiment was performed according to the manufacturer's instructions.
Data analysis	Proteomics data: The default factory settings were used for the Spectronaut Pulsar™ 15.3.210906.50606 (Biognosys, Swiss) search and library generation (including trypsyin/P as the enzyme, up to two missed cleavages allowed Oxidation of Me as a variable modification, carbamidomethyl as a fixed modification, and 1% FDR for PSM, peptide, and protein identification). The DDA search results were imported into Spectronaut Pulsar™. The DIA data were analyzed with Spectronaut to search the above constructed spectral library. The main parameters of the software were set as follows: the precursor Q-value cutoff and protein Q-value cutoff were set as 0.01, the Normalization

Strategy was set as Local Normalization, and MS2 was used as Quantity MS-Level.
The thresholds of fold change (>1.2 or <1/1.2) and p-value (P) < 0.05 and q-value <0.25 were used to identify DEPs. All identified proteins were annotated using GO (http://www.blast2go.com/b2ghome; http://geneontology.org/) and Kyoto Encyclopedia of Genes and Genomes (KEGG) pathway analyses (http://www.genome.jp/kegg/). Differentially expressed protein (DEP)s were used further for GO and KEGG enrichment analyses. Protein-protein interaction analysis was performed using the String (https://string-db.org/) software.

Single-cell sequencing: Single-cell expression data generation
The FastQC software was used to evaluate the data obtained to ensure the quality of the raw sequencing data. The raw data were mapped to the human reference genome (GRCh38, https://cf.10xgenomics.com/supp/cell-exp/refdata-gex-GRCh38-2020-A.tar.gz) using CellRanger, which is a 10x genomics software that labels different mRNA molecules within each cell by identifying the barcode and UMI sequences for single-cell transcriptomic quantification.
Single-cell immune repertoire data generation
The UMI screening standard was supported by 400 paired reads. The reads that passed the mapping and UMI standards were used for contig splicing. Validity screening was also conducted on the barcodes and annotations were provided to remove the error information caused by artificial products. The concatenated contig was annotated and screened further to obtain a consensus sequence supported by the sample CDR3. Clonotype typing was performed based on CDR3 amino acid sequences of the obtained samples with consistent sequences.
Single-cell data analysis
Single-cell data were integrated and clustered using the Seurat R package (version 4) (https://satijalab.org/seurat/). A total of 124,541 cells were obtained from single-cell sequencing of the nine samples, and 108,306 cells remained after quality control. The cell quality control was conducted as follows: cells with a mitochondrial gene ratio exceeding 10% were removed, and only cells with gene numbers ranging from 500 to 4,500 and UMI numbers ranging from 800 to 16,000 were retained. DoubletFinder R package (https://github.com/chris-mcginnis-ucsf/DoubletFinder) was used to remove potential doublets, and further manually remove potentially marginalized doublets based on known classic markers. The filtered data were then standardized and normalized, and principal component analysis was performed on the top 2,000 genes with the highest coefficients of variation. The Harmony R package (https://github.com/immunogenomics/harmony) and the anchor module of Seurat were used to remove inter-batch effects between the samples and groups for cell clustering. Based on the elbow point and significance of the different principal components, the top 30 PCs were selected for subsequent cell clustering, and different resolutions were set to determine the cell clusters. Dimensionality reduction and visualization of single cells were performed using the Uniform Manifold Approximation and Projection (UMAP). The specific execution process of Seurat can be found on the website tutorial (https://satijalab.org/seurat/v4.0/pbmc3k_tutorial.html).
Cell type annotation
Using UMAP, all cells underwent dimensional reduction and were clustered in a two-dimensional space based on shared features. Firstly, the Azimuth algorithm was used to map the data to the reference cell set of PBMC, and then combined with specific high expression genes to manually determine the cell type. Specifically, classic biomarkers for specific cell types were used to identify the cells in different clusters. The FindAllMarkers function in Seurat was used to identify the 50 most highly expressed genes in each cluster of cells, providing a comprehensive understanding of cell types based on the top gene and literature. When clustering for the first or the second time, clusters expressing two or more classic markers and marginalized cells were considered doublets and excluded from subsequent analysis.
Cell difference abundance analysis
Use Milo algorithm to divide the cells of the control group and BA.2-BTI-6m group into different neighborhoods and calculate their spatial distribution differences, mapping them to different cell types. The key parameters for executing the Milo algorithm are k=10 and d=30. In addition, the proportion of cell types for each sample was calculated based on the conventional cell percentage and their differences between groups were calculated using the rank sum test.
Differential gene identification and functional analysis
The Findmarkers() function in the Seurat package was used to identify differentially expressed gene (DEG)s between distinct cell groups, using a standard of |logFC| > 0.25 and FDR < 0.01. DEGs only contain genes expressed in at least 25% of cells of the control group or infection group. The ClusterProfiler R package facilitated Gene Ontology and KEGG enrichment analyses and visualization of DEGs.
Gene set activity score of individual cells
The AddModuleScore() function of Seurat was used to calculate the activity scores of different gene sets in single cells. The gene set was sourced from the msigdb R package (Antigen processing and presentation (hsa04520), JAK_STAT_signaling (hsa04630), B cell activation (GO:0042113), B cell receptor signaling (GO:0050853), positive regulation of Treg activity (GO:0045591), response interferon (GO:0034341), protein processing (GO:0016485) and coagulation regulation (GO:0007597, GO:0050819, GO:0050820, GO:0050818). T cell toxicity activity was defined by the following gene sets: PRF1, IFNG, GNLY, NKG7, GZMB, GZMA, GZMH, KLRK1, KLRB1, KLRD1, CTSW, and CST7. The tissue specific gene set based on proteomics comes from the research of Gutmann et al. and Li et al.
TCR/BCR analysis
Using human GRCh38 as the reference genome, the Cell Ranger vdj pipeline was used to identify the TCR/BCR clonotype and quantify VDJ gene expression. For TCR, we only retained cells with at least one productive TCRα chain (TRA) or TCRβ chain (TRB) for subsequent analysis. Where a cell had two or more paired TRA or TRB chains, we only retained the one with the highest basal expression. Clonotypes were defined based on their unique CDR3 amino acid sequence, and each unique TRA/TRB/TRA-TRB pair was defined as a clonotype. For BCR analysis, we retained only cells with at least one productive heavy chain (IGH) and IGK/IGL for subsequent analysis. When a cell had two or more paired IGH or IGK/IGL chains, only those with the highest basal expression were retained. Each unique pair IGH-IGK/IGL was defined as a clonotype. The scRepertoire R package (https://github.com/ncborcherding/scRepertoire) was used to analyze the single-cell immune repertoire and calculate the clonal diversity of the samples based on the aroma index. Based on the cell barcode information, clonotypes with TCR or BCR were mapped onto the cell UMAP map.

For manuscripts utilizing custom algorithms or software that are central to the research but not yet described in published literature, software must be made available to editors and reviewers. We strongly encourage code deposition in a community repository (e.g. GitHub). See the Nature Portfolio guidelines for submitting code & software for further information.

Data

Policy information about availability of data

All manuscripts must include a data availability statement. This statement should provide the following information, where applicable:

- Accession codes, unique identifiers, or web links for publicly available datasets
- A description of any restrictions on data availability
- For clinical datasets or third party data, please ensure that the statement adheres to our policy

The single-cell sequencing data generated in this study have been deposited in the Genome Sequence Archive63 database under accession code HRA004484 (https://ngdc.cncb.ac.cn/gsa-human). The raw single-cell sequencing data are protected and restrictedly available due to data privacy laws. The processed single-cell sequencing data are available at the Gene Expression Omnibus database (access number: GSE240694, https://www.ncbi.nlm.nih.gov/geo/query/acc.cgi?acc=GSE240694). The mass spectrometry proteomics data generated in this study have been deposited to the ProteomeXchange Consortium via the iProX partner repository under the accession code PXD044441 (http://proteomecentral.proteomexchange.org). The manuscript did not generate original code and the analysis process link can be found in the manuscript methods or contacted by the authors. Source data are provided with this paper.

Research involving human participants, their data, or biological material

Policy information about studies with human participants or human data. See also policy information about sex, gender (identity/presentation), and sexual orientation and race, ethnicity and racism.

Reporting on sex and gender	For COVID-19 convalescents, the median age was 20 years, ranged from 18 years to 29 years, and 48 of 60 were male. For the Healthy cohort, the median age was 25 years, ranged from 20 years to 41 years, and 15 of 20 were male. Sex, number and age of participants in clinical routine examinations and pseudovirus neutralizing assay were in line with the above information and Table S1 except for missing values. Participants included in Proteomic (n=18, median age=23) and single-cell sequencing (n=9, median age=22) analysis were all male. There were two sexes in our study but the female participants accounted for a small portion. Therefore, no sex analysis was carried out. No compensation was included as the participants were delighted to know their physical condition through the thorough examinations.
Reporting on race, ethnicity, or other socially relevant groupings	All samples were from Chinese COVID-19 convalescents. As follow-up biological samples from Omicron BA.2 breakthrough patients were very precious, so the samples were used based on availability and scientific significance.
Population characteristics	For Omicron BA.2 breakthrough infection(BTI) cohort, 2x Double Ad5-vectored COVID-19 vaccine (Convidecia, CanSino Bio Co., Ltd.)-immunized individuals who experienced mild Omicron BA.2 BTI ~10 months after the last shot were recruited (n=60, 48 males and 12 females, aged 18-29). Samples were collected around the same time of a day in a month at three and six months after BTI to minimize the influence of circadian rhythm (BA.2-BTI-3m and BA.2-BTI-6m groups). For the healthy cohort, individuals who had received 2x Convidecia (n=20, 15 males and 5 females, aged 20-41) were recruited for this study. The controls were confirmed uninfected by nucleic acid testing. Blood samples were collected approximately 12 months after the administration of the second dose.
Recruitment	1) Omicron BA.2 breakthrough infected convalescents were diagnosed according to the Chinese Government Diagnosis and Treatment guideline (2021), RT-PCR results and Sanger sequencing by Chinese Center for Disease Control and Prevention. 2) Healthy people were confirmed uninfected by repeated RT-PCR. All participants agreed to be enrolled. Because follow-up samples from Omicron BA.2 breakthrough infected convalescents were hard to access, we enrolled as many as possible in this study. Samples from healthy people were collected from volunteers. Therefore, no self-selection bias were present.
Ethics oversight	This study was approved by the Ethics Committee of the Institute of Microbiology, Chinese Academy of Sciences (SQIMCAS2022127).

Note that full information on the approval of the study protocol must also be provided in the manuscript.

Field-specific reporting

Please select the one below that is the best fit for your research. If you are not sure, read the appropriate sections before making your selection.

[x] Life sciences [ ] Behavioural & social sciences [ ] Ecological, evolutionary & environmental sciences

For a reference copy of the document with all sections, see nature.com/documents/nr-reporting-summary-flat.pdf

Life sciences study design

All studies must disclose on these points even when the disclosure is negative.

Sample size	The follow-up biological samples from Omicron BA.2 breakthrough patients were very precious, so the sample size was determined by availability, scientific logic and materials. The sample size of clinical analysis was 80, including 60 BA.2 breakthrough infected convalescents and 20 healthy control. The sample sizes for other two analyses were determined accordingly, which were 9 (single-cell sequencing, including 5 BA.2 breakthrough and 4 healthy control), and 18 (proteomics analysis, including 10 convalescents and 8 healthy people).
Data exclusions	No patient was excluded for the current study.

Replication	Each sample from the same group was treated as a biological duplicate. We have matched sex and age for single-cell sequencing and proteomics analysis. The main parameters of the software for proteomics data (Data Independent Acquisition (DIA) technique) were set as follows: the precursor Q-value cutoff and protein Q-value cutoff were set as 0.01, the Normalization Strategy was set as Local Normalization.
Randomization	Randomization is not applicable in this study, as the patients were recruited retrospectively based on the clinical diagnosis and treatment guideline.
Blinding	For all the experiments, the investigators were blinded to group allocation, as well as data analysis. For the statistical analysis, no blinding was undertaken in order to deeply excavate the information contained in the datasets.

Reporting for specific materials, systems and methods

We require information from authors about some types of materials, experimental systems and methods used in many studies. Here, indicate whether each material, system or method listed is relevant to your study. If you are not sure if a list item applies to your research, read the appropriate section before selecting a response.

Materials & experimental systems

n/a	Involved in the study
☒	☐ Antibodies
☐	☒ Eukaryotic cell lines
☒	☐ Palaeontology and archaeology
☒	☐ Animals and other organisms
☒	☐ Clinical data
☒	☐ Dual use research of concern
☒	☐ Plants

Methods

n/a	Involved in the study
☒	☐ ChIP-seq
☒	☐ Flow cytometry
☒	☐ MRI-based neuroimaging

Eukaryotic cell lines

Policy information about cell lines and Sex and Gender in Research

Cell line source(s)	Vero cells were purchased from ATCC (CCL81) and used for pseudotyped virus neutralization assay.
Authentication	After purchasing the cell line from ATCC, we have not done authentication as cell lines in our lab were administered by a professional person and who is responsible for quality control of all cell lines.
Mycoplasma contamination	The Vero cells were not tested for Mycoplasma contamination because it does not affect the result of pseudotyped virus neutralization assay.
Commonly misidentified lines (See ICLAC register)	There was no misidentified line used in this study.
